# Evaluation and AI-powered prediction of the efficacy of alirocumab use among patients with cardiovascular disease

**DOI:** 10.3389/fphar.2026.1870914

**Published:** 2026-07-29

**Authors:** Hadeer Ehab Barakat, Ayman Selim Abougalambou, Walid Maher, Samar M. Nour, Islam Tharwat Abdelhalim, Ammena Y. Binsaleh, Dina Mohamed I. Belal, Salwa Selim Abougalambou

**Affiliations:** 1 Department of Clinical Pharmacy Practice, The British University in Egypt, Cairo, Egypt; 2 Royal Commission Health Services Program (RCH) in Aljubail Industrial City, Aljubail, Saudi Arabia; 3 Department of Computer Engineering and Systems, Faculty of Engineering and Technology, Badr University in Cairo (BUC), Cairo, Egypt; 4 School of Information Technology and Computer Science (ITCS), Nile University, Giza, Egypt; 5 Center for Informatics Science (CIS), Nile University, Sheikh Zayed, Egypt; 6 Department of Pharmacy Practice, College of Pharmacy, Princess Nourah Bint Abdulrahman University, Riyadh, Saudi Arabia; 7 Clinical Pharmacy Department, Faculty of Pharmacy (Girls), Al-Azhar University, Cairo, Egypt; 8 Department of Clinical Pharmacy and Pharmacy Practice, Ahram Canadian University, Giza, Egypt

**Keywords:** alirocumab, artificial intelligence, cardiovascular diseases, hyperlipidemias, low-density lipoprotein cholesterol, machine learning, PCSK9 inhibitors

## Abstract

**Background:**

Elevated low-density lipoprotein cholesterol (LDL-C) is a major risk factor for atherosclerotic cardiovascular disease (ASCVD). Despite statins and ezetimibe, many high-risk patients fail to reach lipid targets. Alirocumab, a PCSK9 inhibitor, is an effective alternative, but real-world data are limited.

**Objectives:**

This study evaluated the real-world effectiveness of Alirocumab in improving lipid profiles among patients with established cardiovascular disease and assessed the impact of patient characteristics, comorbidities, and concurrent medications. Additionally, artificial intelligence (AI) models were explored to predict treatment outcomes.

**Methods:**

A retrospective observational study was conducted at the Royal Commission Health Services Program in Aljubail, Saudi-Arabia. Data from 206 adults receiving Alirocumab (75 mg every 2 weeks) from July 2021 to July 2023 were analyzed. Lipid profiles were assessed at baseline, 12 weeks, and 24 weeks. Logistic regression identified factors associated with lipid target achievement. Machine learning models including Logistic-Regression, K-Neighbors, XGBoost, Support Vector Machine (SVM), Gaussian Naive Bayes, Decision Tree, and Random Forest were used to predict outcomes, and an AI-based SVM tool was developed as an exploratory research prototype.

**Results:**

Alirocumab significantly reduced LDL-C, total cholesterol, and triglycerides while increasing HDL-C at 12 and 24 weeks (p < 0.001). Ezetimibe and metformin improved lipid outcomes, whereas aspirin and anticoagulants were associated with lower target achievement. AI model accuracies ranged from 0.50 to 0.63, with the SVM classifier performing best (accuracy 0.625, F1 score 0.61).

**Conclusion:**

Alirocumab is effective in lowering lipids in real-world cardiovascular patients. Certain medications may enhance outcomes. AI models provide moderate predictive ability, supporting potential personalized treatment strategies.

## 1 Introduction

Hypercholesterolemia is a significant risk factor for atherosclerosis and coronary artery disease (CAD), stroke, peripheral vascular disease and the development of cardiovascular complications (Stamler et al., 1986; Wadhera et al., 2016).

An atherogenic lipid profile is characterized by high levels of total cholesterol (TC), low-density lipoprotein cholesterol (LDL-C), and triglycerides (TG), alongside lower-than-normal levels high density lipoprotein cholesterol (HDL-C) (Castelli et al., 1983; Catapano et al., 2016). Given this, reducing LDL-C levels is a crucial approach for both the primary and secondary prevention of atherosclerotic cardiovascular disease.

Statin therapy, either alone or in combination with ezetimibe, is widely regarded as an effective approach for lowering LDL-C levels. However, some patients fail to achieve their target levels despite receiving the maximum tolerated doses of statins and ezetimibe. This may be due to excessively high LDL-C levels or intolerance to these medications caused by adverse side effects.

Alirocumab, a human monoclonal antibody targeting proprotein convertase subtilisin–kexin type 9 (PCSK9) has proven efficacy and safety in improving cardiovascular outcomes after an acute coronary syndrome in patients receiving high-intensity statin therapy.

The European Society of Cardiology (ESC) and the European Atherosclerosis Society (EAS) recommend adding a PCSK9 inhibitor for individuals at very high cardiovascular risk who are unable to reach their LDL-C target (<55 mg/dL or 1.4 mmol/L) despite being on statins and ezetimibe. Likewise, for adults with heterozygous familial hypercholesterolemia (HeFH) whose LDL-C remains at or above 100 mg/dL (2.6 mmol/L) while on the same treatment, a PCSK9 inhibitor is also advised to help further lower cholesterol levels and reduce cardiovascular risk (Mach et al., 2025).

Despite their demonstrated efficacy, the implementation of PCSK9 inhibitors in routine clinical practice remains influenced by factors such as reimbursement policies, treatment costs, and healthcare accessibility, which may limit their use in eligible high-risk patients (Fogacci et al., 2022; El Din Taha et al., 2024). Recent advances in lipid-lowering therapy, including newer agents such as inclisiran and bempedoic acid alongside PCSK9 inhibitors, have expanded treatment options for patients with dyslipidemia. Nevertheless, real-world evidence on the effectiveness of established therapies such as alirocumab remains essential to inform clinical practice (Saad Alghasab et al., 2024).

The odyssey outcomes trial found that patients with a history of acute coronary syndrome whose lipid levels remained high despite taking high-intensity or maximum tolerated doses of atorvastatin or rosuvastatin, treatment with Alirocumab was associated with a lower risk of major cardiovascular events. These included death from coronary heart disease, non-fatal myocardial infarction, fatal or non-fatal ischemic strokes, and hospitalizations due to unstable angina, compared to those who received a placebo (Schwartz et al., 2018).

As these treatments are still fairly new, additional real-world evidence is needed to evaluate their efficacy, safety, and tolerability. A recent review and meta-analysis of 67 studies, involving over 28,000 patients treated with PCSK9 inhibitors in real-world settings, showed an average LDL cholesterol reduction of 87 mg/dL and a 54% drop from baseline levels. This was observed over an average follow-up period of about 9 months, with only 11% of patients discontinuing the treatment. These results are as good as, or even better than, what was seen in controlled clinical trials, where LDL cholesterol dropped by around 60 mg/dL with Alirocumab (Mulder et al., 2022).

The work of Jackevicius et al. (2019) exemplifies the integration of AI-powered prediction models in this domain, emphasizing the prevalence of efficacy and safety prediction models and their potential to improve clinical decision-making. Such models, which incorporate performance criteria such as the C-statistic, should help identify patients who are most likely to benefit from Alirocumab medication while minimizing side effects. Jackevicius et al. (2019) examined various prediction models for efficacy and safety outcomes, noting that performance measures such as the C-statistic were commonly reported, which could facilitate personalized treatment strategies.

Given these findings, this study aims to analyze the demographic and clinical characteristics of patients receiving Alirocumab in real-world settings, assess the cholesterol drop with the use of Alirocumab and the associated factors affecting its effectiveness in lowering LDL-C and achieving target level.

## 2 Methods

### 2.1 Study design

The current study is a retrospective, observational single-arm study conducted at the Royal commission health services program in Aljubail Industrial city, Aljubail, Saudi Arabia.

The current study included all patients who were initiated on treatment with Alirocumab for management of their LDL-C, between July 2021 till July 2023. The primary endpoint of the study is to assess real world reduction in LDL-C associated with the use of Alirocumab and the factors affecting this reduction of LDL-C to reach target level in patients receiving Alirocumab. LDL-C at baseline was compared with LDL-C at 12 weeks and 24 weeks. The secondary outcomes include assessment of the other lipid profile parameters and the factors affecting their reduction including TC, TG and the increase in HDL-C.

Electronic health records of all eligible patients who received Alirocumab were reviewed, and basic demographics as well as patient characteristics at baseline were collected. Additionally, medical history of the patients was collected including cardiovascular history and those patients with hypertension, heart failure, arrhythmias, confirmed coronary artery disease (CAD), peripheral artery disease, stroke, and diabetes mellitus. Furthermore, current medications for their comorbid conditions were also collected and categorized. Each patient’s lipid panel at baseline, and at 12- and 24-weeks post-treatment initiation, also was collected. All the data are collected and tabulated on a predesigned table prior to commencing the study.

### 2.2 Eligibility criteria

The study included all patients ≥18 years diagnosed with cardiovascular disorder and receiving Alirocumab treatments 75 mg every 2 weeks for management of their LDL-C. All patients experienced inadequate control of lipids: LDL-C ≥70 mg/dL (1.8 mmol/L) despite receiving statin and/or other lipid lowering agents.

Patients were excluded from the trial if they received anti-PCSK9 mAb other than Alirocumab, patients with Liver transaminases >3 × ULN, hepatitis B or C infection, creatine kinase >3 × ULN, or patients with eGFR <30 mL/min/1.73 m.

### 2.3 Study outcomes

The primary outcome of the study is to evaluate the efficacy of Alirocumab on lipid profile in patients with CVD and assessments of factors influencing its activity. The evaluation was based on the reduction of LDL-C and other lipid profile from baseline to week 12 and week 24 after receiving Alirocumab treatment.

According to the ESC/EAS guidelines, the LDL-C treatment goal target for very high-risk patients is < 1.4 mmol/L (<55 mg/dL), while the target for high-risk patients is < 1.8 mmol/L (<70 mg/dL).

For other lipid parameters, target levels were defined as total cholesterol <5.2 mmol/L (<200 mg/dL), triglycerides <1.7 mmol/L (<150 mg/dL), and HDL-C >1.0 mmol/L (>40 mg/dL) for males and >1.3 mmol/L (>50 mg/dL) for females.

### 2.4 Ethical considerations

This study is conducted in accordance with the ethical principles originated in the Declaration of Helsinki and that are consistent with Good Clinical Practices and applicable regulatory requirements. Approval of the local ethics committee, Royal Commission for Jubail and Yanbu hospital is granted prior to commencing the study (IRB-RCH-65). Written informed consent was obtained from all participants prior to participation in the study.

### 2.5 Statistical analysis

The data of this study was analyzed by using Statistical Package for Social Science (SPSS) version 26.0 software program. Baseline patient demographics and clinical characteristics were analyzed with frequencies and percentages. For categorical data, using the Chi-Squared test or Fisher’s exact test. For continuous data were represented using independent t-test or Mann-Whitney U test as suitable. Univariate binary logistic regression was conducted to evaluate the association between individual patient characteristics, comorbidities, and concomitant medications and achievement of lipid targets following Alirocumab use. P-value (≤0.05) is considered statistically significant with a confidence interval of 95%.

### 2.6 Artificial intelligence assessment

To assess the predictive performance of various machine learning models, the study followed a structured methodological framework involving data preprocessing, exploratory analysis through a correlation matrix, and the implementation of 7 machine learning models: Logistic Regression, K-Neighbors Classifier, XGBoost, Support Vector Machine (SVM), Gaussian Naive Bayes (GaussianNB), Decision Tree Classifier, and Random Forest. The selected machine learning models were chosen to represent a diverse set of classification paradigms, including linear models (Logistic Regression), distance-based methods (K-Nearest Neighbors), probabilistic approaches (Gaussian Naïve Bayes), kernel-based methods (Support Vector Machine), tree-based learners (Decision Tree), and ensemble methods (Random Forest and XGBoost). This selection allows for a comprehensive comparison of different learning strategies in handling the classification of LDL cholesterol response in high-risk patients. The machine learning models were implemented using Python in the Google Colab environment. The experiments were conducted using Python version 3.11.

The machine learning task was formulated as a supervised classification problem in which the target variable was LDL improvement status. Predictor variables consisted exclusively of patient demographic, clinical, and laboratory characteristics available prior to outcome assessment. The AI component as a proof-of-concept demonstration intended to investigate the feasibility of applying machine learning techniques to the dataset and identify potential predictive patterns.

#### 2.6.1 Data source

The dataset used in this study included clinical and pharmacological data for the 206 patients with cardiovascular disease who received Alirocumab therapy. The dataset contained demographic variables (e.g., body mass index [BMI] and smoking status), comorbidities (such as diabetes mellitus [DM] and hypertension [HTN]), clinical histories (e.g., acute coronary syndrome and coronary revascularization procedures), and concurrent medications including antihypertensive, antidiabetic, and lipid-lowering agents. The primary outcome variable was LDL improvement, representing treatment efficacy. Given the relatively small dataset size (n = 206), model complexity was carefully controlled to reduce overfitting risk. This was achieved through hyperparameter constraints such as limiting tree depth in Decision Tree and Random Forest models, restricting the number of estimators in ensemble methods, and applying regularization in linear models.

#### 2.6.2 Data preprocessing

**FIGURE 1:**
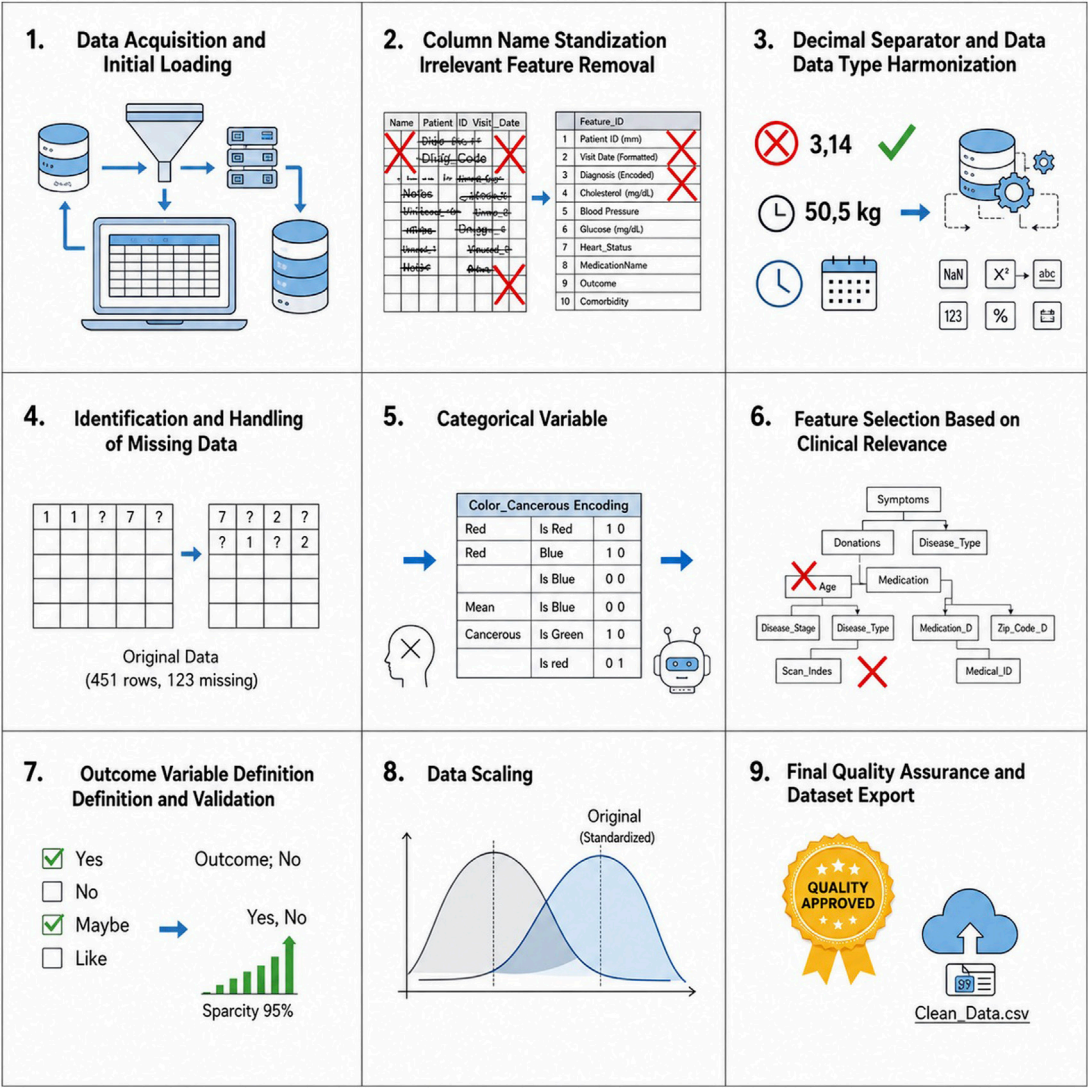
Standardized preprocessing of cardiovascular clinical data: a reproducible framework for AI applications.

The following is a detailed step-by-step guide on creating and preprocessing your dataset for the Alirocumab cardiovascular trial. Each step includes a purpose, action, and justification, making it excellent for methods in the proposed paper as shown in Figure 1.

- Step 1: Data acquisition and initial loading

The study began with a raw dataset containing clinical and pharmacological records of 206 patients diagnosed with cardiovascular disease and treated with Alirocumab. This dataset was stored in a structured format (e.g., CSV or Excel file) and imported into a Python environment using the pandas library. The low_memory = False parameter was used during loading to prevent type inference errors that commonly occur with heterogeneous data columns. At this stage, no modifications were made to the data; the goal was to preserve the original structure for auditability and traceability.

- Step 2: Column name standardization and irrelevant feature removal

Upon inspection, the dataset contained extraneous columns often labeled “Unnamed: 0,” “Unnamed: 1,” etc.—typically generated when saving DataFrames with indices. These columns were removed immediately as they carried no clinical meaning. Additionally, all column names were stripped of leading and trailing whitespace using Python’s.str.strip () method to ensure consistency. This step eliminated potential mismatches during feature selection and improved code readability. For example, a column originally named “BMI” became “BMI”.

- Step 3: Decimal separator and data type harmonization

In some international datasets, decimal values may use commas (e.g., “25,6” for BMI) instead of periods. To ensure uniform numeric interpretation, all known numeric fields (e.g., BMI, baseline LDL, follow-up LDL) were scanned for comma-based decimals. These were systematically replaced with periods using string replacement, after which the columns were converted to floating-point numbers via pd. to_numeric (). Any non-convertible entries (e.g., text like “N/A” or “missing”) were coerced to NaN to flag them for subsequent handling. This step was critical for enabling mathematical operations and statistical modeling.

- Step 4: Identification and handling of missing data

Missing data were addressed with a clinically informed strategy. First, a list of critical variables was defined those essential for analysis, including LDL improvement (the primary outcome), BMI, Age, Sex, HTN, and DM. Any patient record with missing values in one or more of these fields was excluded from the analysis to avoid bias from imputed or incomplete core predictors. For non-critical variables (e.g., details about specific medication brands), missingness was tolerated or handled via mode/median imputation if needed later. This conservative approach prioritized data quality over sample size retention.

- Step 5: Categorical variable encoding

Categorical variables were transformed into numerical formats suitable for machine learning algorithms. For binary variables like hypertension (HTN), custom mapping rules were applied based on clinical documentation. For instance:

“1 controlled” → 1

“0 uncontrolled” or “no” → 0

Ambiguous or invalid entries → NaN (and later excluded)

Similarly, smoking status was encoded ordinally: 0 = never smoked, 1 = former smoker, 2 = current smoker. Medication use (e.g., statin therapy) was treated as binary (1 = yes, 0 = no). All encoding decisions were documented in a codebook to ensure transparency and reproducibility.

- Step 6: Feature selection based on clinical relevance

Not all collected variables were included in modeling. A subset of predictors was selected through consensus among clinicians and data scientists, focusing on factors with established pathophysiological links to lipid metabolism and cardiovascular outcomes. The outcome variable (LDL improvement) and any variables directly derived from it were excluded from the feature-selection process and from all model-training stages. Retained features included:

Demographics: Age, Sex, BMI

Comorbidities: HTN, DM

Clinical history: Acute coronary syndrome (yes/no), prior revascularization

Concurrent medications: Use of antihypertensives, antidiabetics, statins

Variables deemed irrelevant (e.g., hospital ID, date of visit without temporal modeling) or redundant (e.g., duplicate lab entries) were dropped. This reduced noise and improved model interpretability.

- Step 7: Outcome variable definition and validation

The primary outcome, LDL improvement, was explicitly defined—typically as the percent reduction in LDL cholesterol from baseline to follow-up (e.g., ((LDL_baseline - LDL_followup)/LDL_baseline) * 100). This derived variable was computed only for patients with valid measurements at both time points. Its distribution was visualized using histograms and boxplots (matplotlib/seaborn) to check for outliers or skewness. Extreme values (>3 SD from the mean) were reviewed for data entry errors before being retained or corrected.

- Step 8: Data scaling

Although tree-based models (e.g., Random Forest) do not require feature scaling, algorithms like logistic regression, SVM, or neural networks benefit from normalized inputs. For such analyses, numeric features (e.g., Age, BMI, LDL_improvement%) were standardized using StandardScaler from scikit-learn, which transforms data to have a mean of 0 and standard deviation of 1. Importantly, scaling parameters (mean, std) were fitted only on the training set to prevent data leakage during cross-validation.

- Step 9: Final quality assurance and dataset export

Before modeling, the preprocessed dataset underwent final validation:

Confirmed zero missing values in critical columns

Verified data types (e.g., all predictors numeric or binary)

Checked for duplicate patient records

Ensured outcome range was biologically plausible (e.g., LDL improvement between −20% and 80%)

The cleaned feature matrix (X) and outcome vector (y) were saved as separate CSV files (X_preprocessed.csv, y_outcome.csv) for reproducibility and sharing with collaborators.

This meticulous, multi-stage preprocessing pipeline ensured that the resulting dataset was clean, consistent, clinically meaningful, and ready for statistical or machine learning analysis while maintaining transparency and adherence to best practices in biomedical data science.

#### 2.6.3 Feature selection

Twenty-eight potential features were initially defined, covering major cardiovascular risk factors and drug exposure variables. Based on the statistical analysis model and the correlation matrix to measure the relationship between each feature and the target, so only the features present in the dataset were retained, resulting in a subset that included: smoking, BMI, DM, HTN, acute coronary syndrome, coronary revascularization procedure, Aspirin, and Ezetimibe, among others. The dependent variable, *LDL improvement*, served as the target label, whereas all other variables were treated as predictors.

#### 2.6.4 Exploratory data analysis

Descriptive statistics and visualizations were performed to explore the data distribution and relationships between variables. A Pearson correlation matrix was computed using numeric features and visualized via a heatmap (Seaborn library) to identify potential collinearity among predictors and to provide preliminary insight into associations between clinical factors and LDL response.

#### 2.6.5 Model implementation

To predict the LDL improvement (Yes/No), representing Alirocumab efficacy, multiple supervised machine learning algorithms were developed and compared. The focus of the AI model implementation is not on estimating the exact LDL reduction value, but rather on classifying patients according to their treatment response levels. Data was divided into training (80%) and testing (20%) subsets using a stratified random split to preserve outcome balance. Prior to model fitting, features were standardized using the StandardScaler method to ensure uniform scaling and improve model convergence. Table 1 presents a summary of machine learning classifiers with selected hyperparameters and corresponding hyperparameter tuning search space. Hyperparameters were optimized using grid search with stratified k-fold cross-validation. A predefined search space was defined for each model based on commonly used literature values, and the optimal configuration was selected based on mean cross-validation performance.

**TABLE 1:** Summary of machine learning classifiers with selected hyperparameters and corresponding hyperparameter tuning search space.

Model	Key hyperparameters/Settings	Hyperparameter search space
Logistic Regression	max_iter = 1000	C: [0.01, 0.1, 1, 10, 100]; penalty = L2
K-Nearest Neighbors (KNN)	n_neighbors = 5	n_neighbors: (Castelli et al., 1983; Mach et al., 2025; El Din Taha et al., 2024; Schwartz et al., 2018; Jackevicius et al., 2019); weights: [uniform, distance]
XGBoost Classifier	n_estimators = 100, learning_rate = 0.1, max_depth = 3	n_estimators: [50, 100, 200]; learning_rate: [0.01, 0.1, 0.2]; max_depth: (Castelli et al., 1983; Mach et al., 2025; El Din Taha et al., 2024)
Support Vector Machine (SVM)	Kernel = RBF, 5-fold cross-validation	C: [0.1, 1, 10, 100]; gamma: [0.001, 0.01, 0.1, 1]; 5-fold cross-validation used
Gaussian Naïve Bayes	Default parameters	var_smoothing: [1e-9, 1e-8, 1e-7, 1e-6]
Decision Tree Classifier	max_depth = 3	max_depth: [3, 5, 10, None]; min_samples_split: (Wadhera et al., 2016; Mach et al., 2025; Mulder et al., 2022)
Random Forest Classifier	n_estimators = 100, max_depth = 5	n_estimators: [50, 100, 200]; max_depth: [5, 10, None]; min_samples_split: (Wadhera et al., 2016; Mach et al., 2025; Mulder et al., 2022)

KNN = K-Nearest Neighbors; SVM, support vector machine; RBF, radial basis function; max_iter = maximum number of iterations; n_estimators = number of trees in the ensemble; max_depth = maximum tree depth; min_samples_split = minimum number of samples required to split an internal node; C = regularization parameter; gamma = kernel coefficient; learning_rate = learning rate used during model training; var_smoothing = variance smoothing parameter.

#### 2.6.6 Model evaluation metrics

Each model was trained on the scaled training set and evaluated on the test set. Performance metrics included accuracy, precision, recall, F1-score, and the confusion matrix, derived using scikit-learn’s built-in functions. Cross-validation was applied to estimate model robustness and generalization performance.

#### 2.6.7 Model deployment

The application is deployed using Gradio in combination with Hugging Face Spaces. The model and its dependencies are packaged along with the app, and the environment is configured via a requirements. txt file to ensure version compatibility. The app runs in a cloud environment, providing a permanent URL for interactive use, enabling users to input data and receive predictions directly through the browser without any local setup (https://huggingface.co/spaces/Islamhalim/Alirocumab).

## 3 Results

### 3.1 Sociodemographic data and characteristics of patients

Table 2 summarizes the demographic and clinical characteristics of 206 participants. The majority were male (75.2%) and predominantly aged between 41 and 64 years (84%). Most participants were Saudi nationals (93.2%), with a significant proportion reporting no history of smoking (65%). Regarding physical activity, 59.7% were considered non-active. In terms of body mass index (BMI), 44.2% were classified as obese, while 38.3% were overweight. Type 2 diabetes was prevalent among 68% of the patients, and 83% had hypertension. Acute coronary syndrome was reported in various forms, including NSTEMI (33.5%), unstable angina (23.8%), and STEMI (12.6%). Over half (58.7%) underwent percutaneous coronary intervention (PCI), while 6.8% had coronary artery bypass grafting (CABG). Additionally, 44.2% had heart failure, 6.3% had peripheral artery disease (PAD), and 1.5% had a history of stroke or atrial fibrillation.

**TABLE 2:** Demographic data and characteristics of patients.

Variable	N (%)
Gender	
Male	155 (75.2%)
Female	51 (24.8%)
Age (years)	
≤40 years	10 (4.8%)
41–64 years	173 (84%)
≥65 years	23 (11.2%)
Nationality	
Saudi	192 (93.2%)
Other	14 (6.8%)
Smoking history	
Yes	72 (35%)
No	134 (65%)
Alcohol history	
Yes	2 (1%)
No	204 (99%)
Physical activity	
Non active <150 min/week	123 (59.7%)
Active ≥150 min/week	83 (40.3%)
BMI (kg/m^2^)	
Under weight <18.5 kg/m^2^	2 (1%)
Normal weight <25 kg/m^2^	34 (16.5%)
Overweight >25–30 kg/m^2^	79 (38.3%)
Obese ≥30 kg/m^2^	91 (44.2%)
Diabetes mellitus	
Type I Diabetes	2 (1%)
Type 2 Diabetes	140 (68%)
No	64 (31.1%)
Hypertension	
Yes	171 (83%)
No	35 (17%)
Acute coronary syndrome	
Unstable angina	49 (23.8%)
Non-ST-elevation myocardial infarction (NSTEMI)	69 (33.5%)
ST-elevation myocardial infarction (STEMI)	26 (12.6%)
No	62 (29.2%)
Coronary revascularization procedure	
Percutaneous coronary intervention (PCI)	121 (58.7%)
Coronary artery bypass grafting (CABG)	14 (6.8%)
No	71 (34.5%)
Heart failure	
Yes	91 (44.2%)
No	115 (55.8%)
Peripheral artery disease (PAD)	
Yes	13 (6.3%)
No	193 (93.7%)
Stroke	
Yes	3 (1.5%)
No	203 (98.5%)
Atrial fibrillation	
Yes	3 (1.5%)
No	203 (98.5%)
Cardiovascular risk category of the study population according to ESC/EAS risk stratification	
Very High Risk	146 (70.9%)
High Risk	60 (29.1%)
Baseline Lipid profile	Mean (mmol/L) ± SD
LDL-C	3.78 ± 1.41
HDL-C	1.17 ± 0.713
Total cholesterol	5.77 ± 1.54
Triglycerides	2.31 ± 1.62

BMI: body mass index, ESC/EAS: European Society of Cardiology (ESC) and the European Atherosclerosis Society.

### 3.2 Changes in the lipid profile level 12 weeks after alirocumab use

Table 3 presents the mean changes in lipid profile parameters between Visit 1 (V1) and Visit 2 (V2) after 12 weeks of Alirocumab use, including standard deviations, 95% confidence intervals (CIs), and p-values. A statistically significant reduction in LDL levels was observed, with a mean change of −1.25 ± 1.09 (95% CI: −1.41 to −1.08; p < 0.001). HDL levels demonstrated a significant increase of 0.089 ± 0.22 (95% CI: 0.056 to 0.123; p < 0.001). Likewise, total cholesterol levels declined markedly, with a mean change of −1.73 ± 1.40 (95% CI: −1.93 to −1.52; p < 0.001). Triglyceride levels also showed a significant decrease of −0.73 ± 1.39 (95% CI: −0.951 to −0.513; p < 0.001). These findings confirm that notable and favorable changes in lipid profiles occurred as early as the second visit following Alirocumab initiation.

**TABLE 3:** Mean change of lipid profile after 12 weeks of Alirocumab use.

Variable	Mean after 12 weeks ± SD	Mean change (V1-V2) ± SD	Mean percentage change from baseline	95% confidence interval	P -value
LDL-C	2.53 ± 1.22	−1.25 ± 1.09	−33.1%	(-1.41 to −1.08)	<0.001*
HDL-C	1.25 ± 0.33	0.089 ± 0.22	+7.6%	(0.056 to 0.123)	<0.001*
Total Cholesterol	4.04 ± 0.89	−1.73 ± 1.40	−30.0%	(-1.93 to −1.52)	<0.001*
Triglyceride	1.58 ± 0.75	−0.73 ± 1.39	−31.6%	(-0.951 to −0.513)	<0.001*

* Significant, pvalue <0.001.

### 3.3 Changes in the lipid profile level 24 weeks after alirocumab use

Table 4 presents the mean changes in lipid profile parameters between Visit 1 (V1) and Visit 3 (V3), along with their standard deviations, 95% confidence intervals (CIs), and associated p-values. There was a statistically significant decrease in LDL levels, with a mean change of −2.19 ± 1.25 (95% CI: −2.377 to −2.002; p < 0.001). Conversely, HDL levels showed a significant increase, with a mean change of 0.22 ± 0.35 (95% CI: 0.165 to 0.268; p < 0.001). Total cholesterol also decreased significantly by −2.66 ± 1.63 (95% CI: −2.906 to −2.421; p < 0.001). Additionally, triglyceride levels decreased by −0.95 ± 1.88, with a 95% CI ranging from −1.238 to −0.666. These results suggest notable alterations in lipid profiles over the course of the study.

**TABLE 4:** Mean change of lipid profile after 24 weeks of Alirocumab use.

Variable	Mean after 24 weeks ± SD	Mean change (V1-V3) ± SD	Mean percentage change from baseline	95% confidence interval	P -value
LDL-C	1.59 ± 0.88	−2.19 ± 1.25	−57.9%	(−2.377 to −2.002)	<0.001*
HDL-C	1.38 ± 0.34	0.22 ± 0.347	+18.8%	(0.165 to 0.268)	<0.001*
Total Cholesterol	3.11 ± 1.91	−2.66 ± 1.63	−46.1%	(−2.906 to −2.421)	<0.001*
Triglyceride	1.36 ± 1.24	−0.95 ± 1.88	−41.1%	(−1.238 to −0.666)	<0.001*

* Significant, pvalue <0.001.

### 3.4 Patient related factors and comorbidities affecting the efficacy of alirocumab on the lipid profile of the patients after 24 weeks

As shown in Table 5, the logistic regression analysis evaluated how patient related factors and comorbidities influenced the odds of achieving lipid profile targets with Alirocumab treatment. BMI was significantly associated with triglyceride target achievement, where each unit increase in BMI slightly increases the odds of achieving the target (OR = 1.057, 95% CI: 1.000–1.118, *p* = 0.048). Surprisingly, smoking was also significantly associated with triglyceride outcomes, with smokers having significantly decreased odds of achieving target triglyceride levels compared to non-smokers (OR = 0.36, 95% CI: 0.182–0.714, *p* = 0.003). Other associations between BMI and LDL, HDL, or total cholesterol targets were not statistically significant. Additionally, diabetes mellitus, hypertension, and acute coronary syndrome did not show significant effects on achieving any lipid targets with Alirocumab. Although coronary revascularization (PCI or CABG) showed reduced odds of achieving lipid control, these associations were not statistically significant.

**TABLE 5:** Patients-related factors and comorbidities affecting the efficacy of Alirocumab.

Item	LDL target						HDL target						Total cholesterol target						Triglyceride target					
	Yes	No	OR	95% CI		P - value	Yes	No	OR	95% CI		P - value	Yes	No	OR	95% CI		P - value	Yes	No	OR	95% CI		P - value
BMI	​	​	1.015	0.963	1.070	0.584	​	​	1.03	0.97	1.094	0.333	​	​	1.027	0.972	1.085	0.338	​	​	1.057	1	1.118	0.048*
Smoking	80	54	0.909	0.504	1.641	0.752	33	101	1.319	0.63	2.772	0.465	81	53	1.173	0.614	2.242	0.63	66	68	0.36	0.182	0.714	0.003*
Diabetes mellitus	83	56	1.015	0.539	1.910	0.963	35	104	1.206	0.58	2.531	0.62	82	57	1.048	0.548	2.001	0.888	64	75	1.708	0.88	3.319	0.114
Hypertension	106	66	1.239	0.536	2.861	0.616	43	129	1.387	0.47	4.077	0.552	103	69	0.939	0.388	2.272	0.889	78	94	1.906	0.723	5.024	0.192
Acute coronary syndrome	88	56	1.497	0.500	4.481	0.47	36	108	1.63	0.49	5.378	0.423	80	64	0.694	0.228	2.11	0.52	64	80	1.95	0.62	6.13	0.253
Coronary revascularization	80	55	0.89	0.5	1.609	0.39	32	103	0.598	0.2	1.839	0.37	73	62	0.567	0.198	1.623	0.29	57	78	0.403	0.134	1.209	0.105

### 3.5 Medication related factors affecting the efficacy of alirocumab on the lipid profile of the patients

As shown in Table 6, through the evaluation of the effect of concomitant medications on achieving lipid targets with Alirocumab, several noteworthy associations were observed. Ezetimibe significantly increased the odds of achieving LDL targets (OR = 3.56, 95% CI: 1.0–12.2, *p* = 0.043), highlighting its positive contribution when combined with Alirocumab. In contrast, aspirin use was significantly associated with reduced odds of achieving LDL (OR = 0.45, CI: 0.2–0.95, *p* = 0.035), total cholesterol (OR = 0.36, 95% CI:0.2–0.8, *p* = 0.01), and triglyceride targets (OR = 0.4, CI: 0.2–0.75, *p* = 0.01). Metformin significantly improved the odds of achieving triglyceride targets (OR = 2.6, 95% CI: 1.2–5.68, *p* = 0.02), suggesting a beneficial synergistic effect with Alirocumab. Similarly, anticoagulants showed a significant reduction in the likelihood of achieving total cholesterol targets (OR = 0.07, 95% CI: 0–0.85, *p* = 0.04). Most other medications, including empagliflozin, sitagliptin, insulin, valsartan, amlodipine, and bisoprolol, did not show statistically significant effects on lipid target attainment, although directional trends varied. While statin appeared to numerically reduce the odds of achieving certain lipid targets, these findings were not statistically significant. Overall, the data suggests that metformin and ezetimibe enhance Alirocumab’s lipid-lowering efficacy, while aspirin and anticoagulants may attenuate its effects on specific lipid fractions.

**TABLE 6:** Medication-related factors affecting the efficacy of Alirocumab.

Item	LDL target						HDL target						Total cholesterol target						Triglyceride target					
	Yes	No	OR	95% CI		P - value	Yes	No	OR	95% CI		P - value	Yes	No	OR	95% CI		P - value	Yes	No	OR	95% CI		P - value
Metformin	51	34	1.22	0.6	2.62	0.610	21	64	1.32	0.6	3	0.51	45	40	0.85	0.4	1.94	0.7	40	45	2.6	1.2	5.68	0.02*
Empagliflozin	32	25	0.84	0.4	1.96	0.685	12	45	0.68	0.3	1.7	0.42	27	30	0.62	0.3	1.53	0.30	24	33	0.8	0.4	1.96	0.68
Sitagliptin	23	14	0.99	0.4	2.52	0.977	8	29	0.75	0.3	2.1	0.58	18	19	0.86	0.3	2.28	0.76	13	24	0.7	0.3	1.71	0.39
Gliclazide	9	8	0.81	0.2	2.67	0.734	3	14	0.78	0.2	3.2	0.73	7	10	0.37	0.1	1.22	0.10	9	8	1.2	0.4	3.73	0.78
Semaglutide	0	4	0	0	​	0.999	0	4	0	0	​	1	0	4	0	0	​	1	3	1	9.4	0.8	116	0.08
Insulin	14	11	0.82	0.3	2.24	0.703	7	18	1.73	0.6	5.1	0.32	14	11	1.11	0.4	3.21	0.84	8	17	0.5	0.2	1.42	0.19
Valsartan	44	25	2.1	0.9	4.86	0.083	20	49	1.57	0.7	3.7	0.31	43	26	1.69	0.7	4.11	0.25	30	39	1.2	0.6	2.72	0.59
Amlodipine	19	12	1.36	0.5	3.63	0.545	9	22	1.32	0.5	3.6	0.59	20	11	1.73	0.6	4.97	0.31	12	19	0.8	0.3	2.08	0.62
Bisoprolol	44	33	0.73	0.3	1.56	0.42	19	58	0.82	0.4	1.8	0.63	39	38	0.69	0.3	1.52	0.36	27	50	0.9	0.4	1.84	0.7
Carvidilol	1	2	0.16	0	2.2	0.169	1	2	0.57	0	8.3	0.68	3	0	​	0	​	1	2	1	2	0.1	28.3	0.61
Perindopril	12	9	1.25	0.4	4.04	0.709	3	18	0.74	0.2	3.2	0.68	9	12	0.63	0.2	2.11	0.45	5	16	0.3	0.1	1.29	0.11
Sacubatril	4	5	0.82	0.1	5.4	0.839	2	7	1.12	0.2	7	0.91	5	4	0.82	0.1	5.14	0.83	4	5	0.9	0.2	4.23	0.87
Sacubatril + valsartan	2	1	-	0	​	0.999	0	3	0	0	​	1	1	2	0.12	0	1.77	0.12	2	1	1.9	0.1	25.1	0.64
Spironolactone	0	5	0	0	​	0.999	0	5	0	0	​	1	2	3	1.44	0.1	19.2	0.78	2	3	1	0.1	9.06	1
Thiazide	7	4	0.66	0.1	2.93	0.583	2	9	0.52	0.1	2.9	0.46	5	6	0.21	0	1.06	0.06	4	7	0.7	0.1	3.12	0.61
Aspirin	56	56	0.45	0.2	0.95	0.035*	25	87	0.96	0.4	2.1	0.92	53	59	0.36	0.2	0.8	0.01*	36	76	0.4	0.2	0.75	0.01*
Clopidogrel	20	24	0.81	0.3	1.97	0.637	10	34	1.1	0.4	3	0.86	17	27	0.53	0.2	1.34	0.18	13	31	1	0.4	2.48	0.94
Ticagrelor	4	6	0.63	0.1	3.06	0.566	1	9	0.29	0	2.8	0.28	3	7	0.23	0	1.14	0.07	3	7	0.9	0.2	4.44	0.86
Anticoagulant	3	2	0.34	0	2.79	0.316	2	3	1.27	0.2	11	0.83	2	3	0.07	0	0.85	0.04*	2	3	0.6	0.1	4.9	0.65
Statin	120	80	0.26	0	2.41	0.236	46	154	0.24	0	2	0.19	120	80	0.06	0	1.93	0.11	83	117	1.2	0.1	10.4	0.89
Ezetimibe	116	73	3.56	1	12.2	0.043*	44	145	1.4	0.4	5.5	0.63	113	76	1.49	0.4	5.45	0.55	78	111	1	0.3	3.23	0.96

* significant, pvalue<0.05, OR: odds ratio, CI: confidence interval.

### 3.6 Artificial intelligence assessment for patient-related factors, comorbidities, and medications affecting the efficacy of alirocumab on the lipid profile of the patients

**FIGURE 2:**
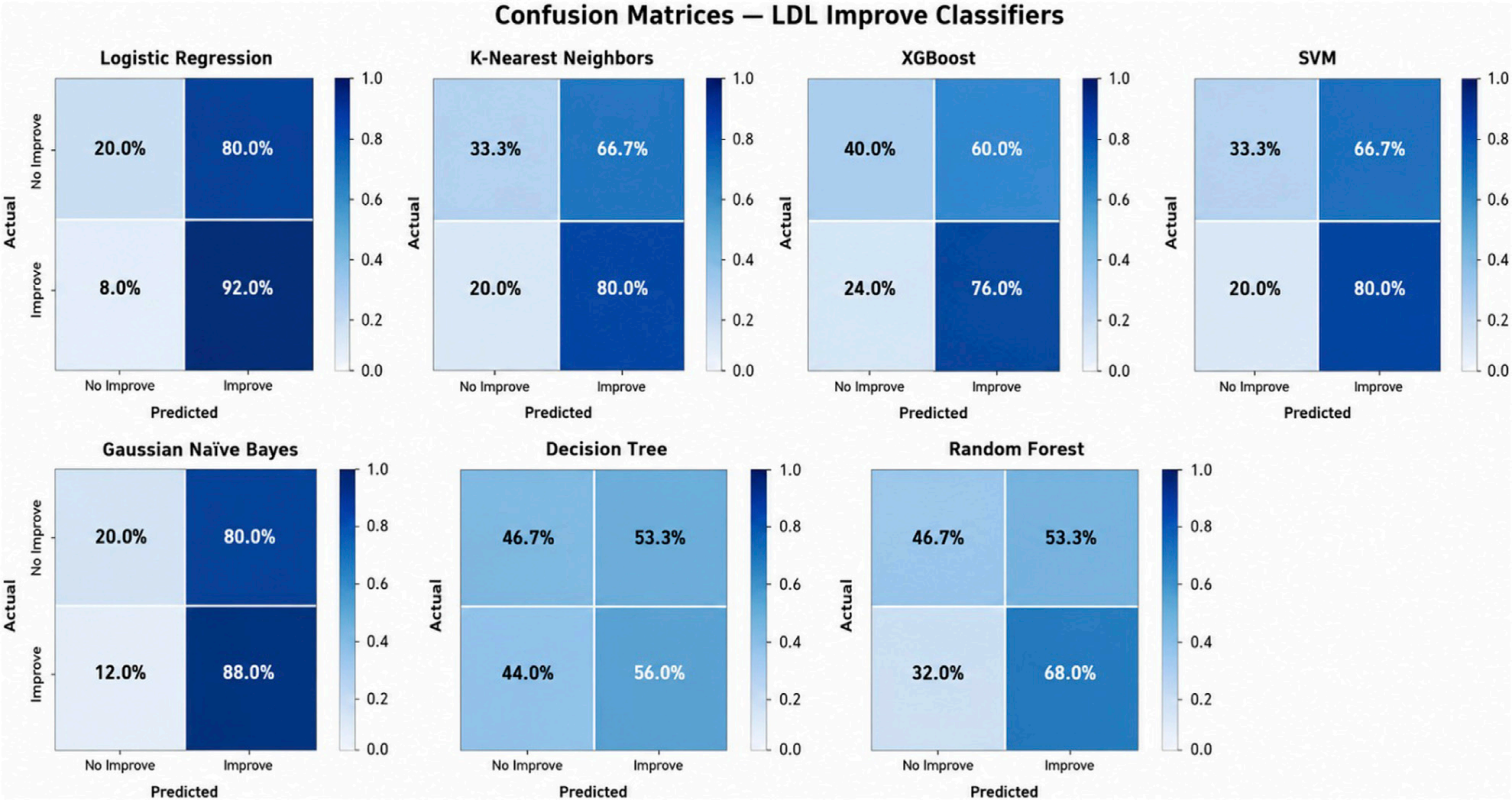
Normalized confusion matrices for LDL improvement classifier.

As shown in Table 7, seven supervised machine learning models were applied and evaluated to predict the improvement of low-density lipoprotein (LDL) levels among patients with cardiovascular disease, indicating Alirocumab efficacy. The models included Logistic Regression, K-Nearest Neighbors (KNN), XGBoost Classifier, Support Vector Machine (SVM), Gaussian Naïve Bayes (GaussianNB), Decision Tree Classifier, and Random Forest Classifier. After data preprocessing and standardization, the models were trained on 80% of the dataset and tested on the remaining 20%. Performance was assessed using standard classification metrics—accuracy, precision, recall, F1-score, and the confusion matrix—to evaluate predictive reliability in distinguishing between patients who showed LDL improvement and those who did not. Results are presented as mean ± standard deviation obtained from cross-validation experiments. Figure 2 presents the normalized confusion matrices of the seven machine learning models used to classify LDL improvement. Overall, all models showed a tendency to predict the “Improve” class, resulting in high sensitivity but lower specificity. Logistic Regression and Gaussian Naïve Bayes achieved the highest true positive rates (92% and 88%, respectively), but also produced the highest false positive rates, misclassifying 80% of the “No Improve” cases. In contrast, Decision Tree and Random Forest reduced false positives to 53.3%, although their ability to correctly identify improvement cases decreased. KNN and SVM provided a more balanced performance, achieving an 80% true positive rate with a 66.7% false positive rate. These findings suggest a trade-off between sensitivity and specificity across all models, likely influenced by class imbalance in the dataset.

**TABLE 7:** Summary of AI Model Performance (mean ± standard deviation).

Variable	Accuracy	Precision (Avg)	Recall (Avg)	F1-score (Avg)
Logistic Regression	0.50 ± 0.05	0.44 ± 0.06	0.50 ± 0.05	0.44 ± 0.05
K-Nearest Neighbors (KNN)	0.57 ± 0.04	0.56 ± 0.05	0.57 ± 0.04	0.55 ± 0.05
XGBoost Classifier	0.58 ± 0.03	0.57 ± 0.04	0.58 ± 0.03	0.57 ± 0.04
Support Vector Machine (SVM)	0.63 ± 0.03	0.62 ± 0.03	0.62 ± 0.03	0.61 ± 0.03
Gaussian Naïve Bayes (GNB)	0.53 ± 0.07	0.32 ± 0.08	0.53 ± 0.07	0.40 ± 0.07
Decision Tree Classifier	0.53 ± 0.06	0.55 ± 0.06	0.53 ± 0.06	0.52 ± 0.06
Random Forest Classifier	0.57 ± 0.03	0.58 ± 0.03	0.57 ± 0.03	0.58 ± 0.03

**FIGURE 3:**
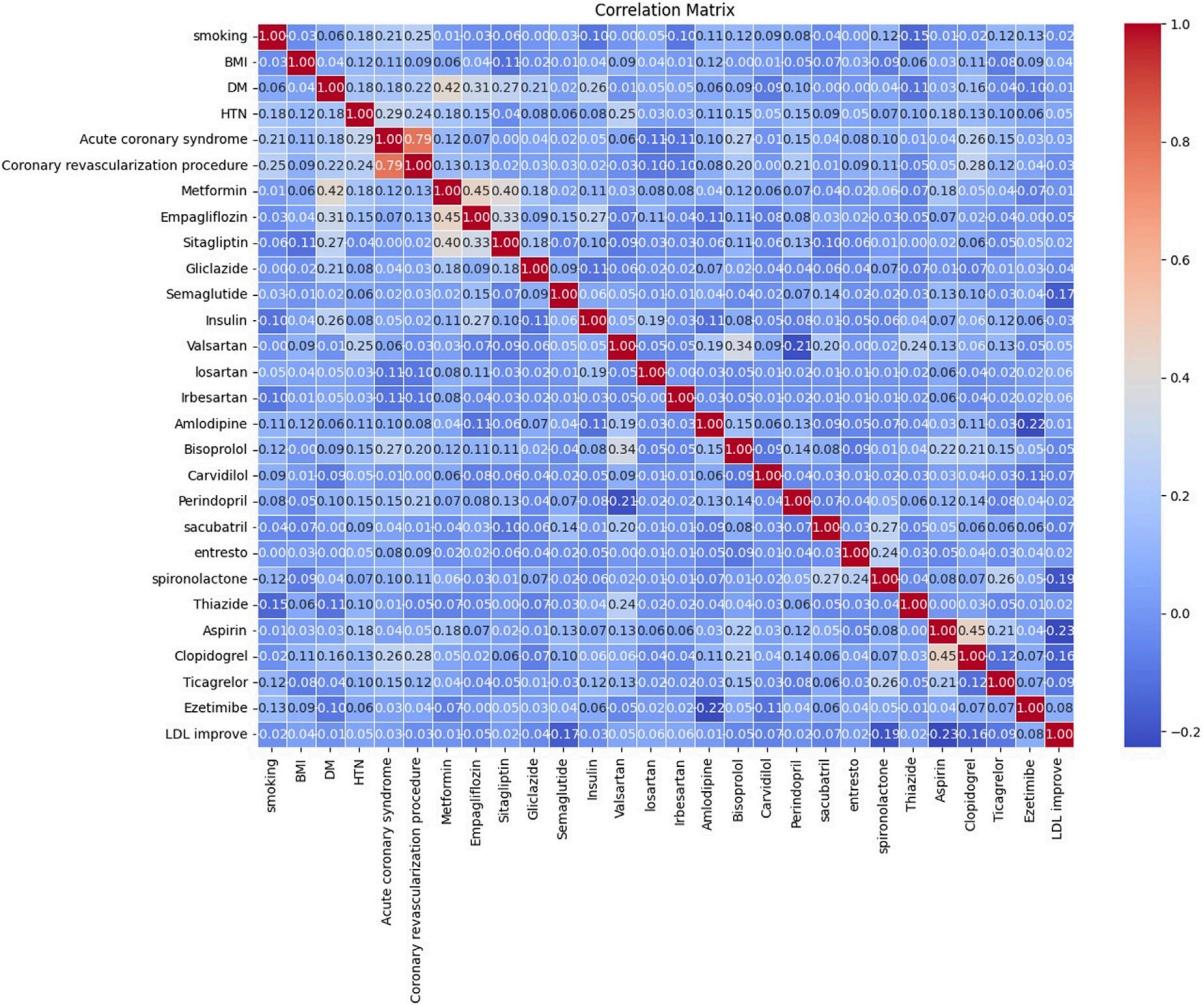
Correlation matrix between patients characteristics and their medication with reduction of LDL cholesterol to target level after 6 months of Alirocumab use.

As shown in Figure 3, the correlation matrix illustrates the relationships between clinical features, medications, and *LDL improvement* among patients using Alirocumab. Overall, most variables show weak to moderate correlations, suggesting that LDL response is influenced by multiple interacting factors rather than a single dominant predictor.

The predictive accuracy across the models ranged from 0.50 to 0.63, indicating moderate discrimination capability. Among all classifiers, the Support Vector Machine (SVM) achieved the highest test accuracy of 0.625, with a balanced performance across both classes. This suggests that nonlinear relationships between clinical variables and treatment response may be best captured by SVM’s kernel-based learning approach. The K-Nearest Neighbors (KNN) and Random Forest models both achieved accuracies of approximately 0.57, demonstrating consistent but moderate predictive power. The Decision Tree Classifier reached an average cross-validated accuracy of 0.53, while Logistic Regression performed slightly lower, with accuracies around 0.50, indicating limited capacity to model complex feature interactions. The XGBoost Classifier, configured with 100 estimators, a learning rate of 0.1, and maximum depth of 3, produced accuracy comparable to Random Forest, confirming the value of ensemble methods but also reflecting potential data limitations. The Gaussian Naïve Bayes model achieved 0.53 accuracy, with a tendency to predict positive LDL improvement but near-zero recall for non-responders, indicating class imbalance sensitivity.

**FIGURE 4:**
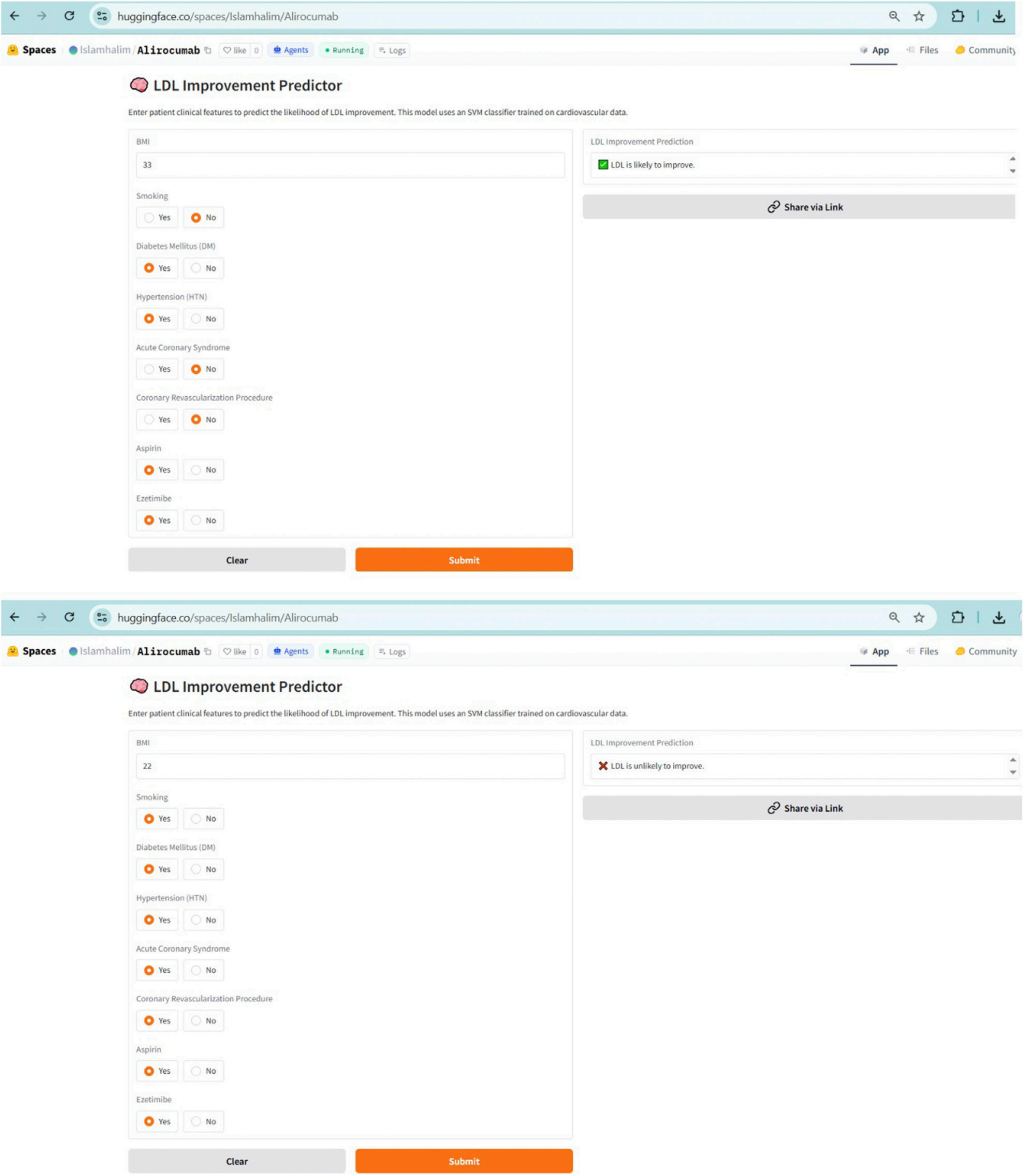
User interface of the LDL Improvement Predictor. Users input clinical features such as BMI, comorbidities, smoking status, and medication use. The application processes the data and provides a prediction of LDL improvement.

Finally, we developed the LDL Improvement Predictor, a web-based application using a trained SVM model to estimate the likelihood of LDL cholesterol improvement from patient clinical features. The developed application is intended solely as an exploratory research prototype to demonstrate the feasibility and potential application of the proposed methodology. The application has not undergone the rigorous clinical validation, regulatory review, or prospective evaluation required for deployment in real-world healthcare settings. As shown in Figure 4, users enter key inputs such as BMI, smoking status, comorbidities, and medication usage. The app automatically processes these inputs, aligns them with the model’s expected features, and provides both a prediction and confidence score. Built with Gradio and deployed on Hugging Face Spaces, it is accessible via a shareable web link.

## 4 Discussion

The current study offers insights into the clinical characteristics and treatment practices of patients diagnosed with established atherosclerotic cardiovascular disease (ASCVD) who were prescribed Alirocumab as part of routine care in Saudi Arabia. In addition to the effect of these factors on their response to Alirocumab.

This research ensures the already established clinical efficacy of Alirocumab in treating patients with raised LDL-C who do not respond to standard lipid-lowering treatments. The outcomes of this study, which demonstrated improvements in LDL-C at 12 weeks and 24 weeks after Alirocumab use are in accordance with results from large clinical trials like odyssey outcomes (Schwartz et al., 2018). In addition to the marked reduction in LDL-C, modest improvements were observed in other lipid parameters, including HDL-C and triglycerides. These results support the clinical effectiveness of PCSK9 inhibitors in managing patients with cardiovascular diseases in real-world clinical settings.

Before commencing Alirocumab, statin therapy (notably atorvastatin or rosuvastatin) was typically employed to manage dyslipidaemia, either as monotherapy or, more commonly, in conjunction with ezetimibe. This aligns with the recommendations which state that patients with hypercholesterolaemia should begin treatment with statins, followed by ezetimibe if necessary (Piepoli et al., 2016).

The significant improvements observed in lipid parameters at 12 weeks post-initiation of Alirocumab provide compelling evidence for its early therapeutic impact in real-world clinical practice. The marked reduction in LDL-C levels by an average of 1.25 ± 1.09 mmol/L (48.34 mg/dL ±42.15 mg/dL) aligns with findings from controlled clinical trials and underscores Alirocumab’s potency in achieving LDL-C targets, particularly in high-risk patients with a history of statin intolerance or inadequate response to prior therapy (Steffens et al., 2021). Moreover, the 24 weeks mean change in LDL-C significantly enhanced by an average reduction of 2.19 mmol/L ±1.25 (84.69 mg/dL ±48.34).

A modest but statistically significant increase in HDL-C was observed; however, its clinical significance is secondary to the substantial LDL-C reduction achieved with alirocumab. Nevertheless, this finding is consistent with previous reports by Shukla et al. and supports an overall improvement in the lipid profile (Shukla and Mehani, 2019). Additionally and in agreement with Zahger et al., reductions in triglycerides was also statistically significant, suggesting that Alirocumab exerts a broad lipid-lowering effect beyond LDL-C (Zahger et al., 2024). These early results reinforce the rationale for incorporating PCSK9 inhibitors such as Alirocumab into treatment regimens for patients not meeting lipid targets with conventional therapy alone, and they highlight the value of monitoring therapeutic outcomes as early as 12 weeks to guide clinical decision-making.

Significant improvements in the lipid profile was found in 24 weeks after Alirocumab use. This study’s Alirocumab treatment also resulted in a mean LDL-C reduction of 2.19 mmol/L, which further corroborates the attaining of recommended LDL levels. The European Society of Cardiology (ESC) and the European Atherosclerosis Society (EAS) supports the 50% reduction of LDL-C in high-risk patients and includes also a goal of <1.8 mmol/L (70 mg/dL) especially in patients with atherosclerotic cardiovascular disease (Feingold, 2025).

The majority of patients were receiving background lipid-lowering therapy, including statins and ezetimibe, prior to Alirocumab initiation then added Alirocumab markedly enhanced their lipid levels. Such results bolster current ESC/EAS Task Force issues updated clinical guidance for the use of PCSK9 inhibitors that call for the use of PCSK9 inhibitors in patients with stubborn high LDL-C levels despite multi-drug therapy (Koskinas et al., 2021).

Patient factors and comorbidities were analyzed to determine whether they impacted achievement of lipid targets. It is noteworthy that body mass index (BMI) showed a positive and statistically significant association with achieving triglyceride goals. This may indicate that higher-BMI patients, who tend to be more metabolically active, may receive a greater therapeutic response from treatment owing to elevated triglyceride levels at baseline. This result is in disagreement with the findings of the pooled analysis of Phase 3 ODYSSEY which showed that Alirocumab provided consistent LDL-C reductions, with similar safety findings across BMI subgroups (Tinahones et al., 2020). Smoking was also linked to decrease the odds of achieving triglyceride targets given the pro-atherogenic effects of smoking.

In contrast, comorbidities including hypertension, diabetes mellitus, acute coronary syndrome, and a history of coronary revascularization did not appear to significantly impact the likelihood of achieving lipid targets. This indicates that there is a predominant consistency in the lipid-lowering efficacy of Alirocumab across varying profiles of cardiovascular disease. These findings support the potential use of Alirocumab in diverse clinical populations, irrespective of the severity of pre-existing cardiovascular disease or metabolic comorbidities.

The study by Toth et al., demonstrated that Alirocumab produced substantial and consistent reductions in LDL-C levels in patients with hypercholesterolemia, regardless of renal function status. LDL-C reduction at week 24 ranged from approximately 46%–62% in patients with chronic kidney disease and 48%–60% in those without impaired renal function (Toth et al., 2018). Similarly the study by Tunon et al., showed that in patients with recent acute coronary syndrome, Alirocumab treatment was associated with a reduction in major adverse cardiovascular events and fewer deaths, independent of baseline estimated glomerular filtration rate, across a broad range above 30 mL/min/1.73 m^2^ (Tunon et al., 2020).

The review of concomitant medications revealed some important overlapping interactions. Ezetimibe markedly increased the probability of reaching LDL-C targets, which is consistent with its synergistic effect with PCSK9 inhibitors (Gunta et al., 2023). This reinforces current clinical approaches which often prefer to use a combination of lipid lowering treatments to achieve optimal outcomes, especially in patients who do not respond to statin therapy. Aspirin use was associated with lower odds of achieving LDL-C, total cholesterol, and triglyceride targets. However, these findings should be interpreted with caution because the observed association could be more likely to reflect confounding by indication, as patients receiving aspirin may have had more advanced atherosclerotic cardiovascular disease or a higher baseline cardiovascular risk profile. Similarly, the association between anticoagulant use and lower total cholesterol target achievement was based on a very small number of patients and should be considered exploratory rather than conclusive. Further studies with larger sample sizes and appropriate adjustment for confounding factors are needed to clarify these observations.

Within the antidiabetic drugs, metformin was associated with a favorable impact on triglyceride reduction in agreement with previous studies (Geerling et al., 2014; Hu et al., 2021). Given the improvement in intrahepatic glucose metabolism and reducing extrahepatic insulin, metformin exerted a beneficial effect on triglycerides metabolism (Xu et al., 2015; Wu et al., 2016). This effect is plausible and indicates an additional benefit with Alirocumab in diabetic patients when metformin is part of the regimen. However, it should be interpreted cautiously, as the observed association may be influenced by diabetes status, baseline triglyceride levels, BMI, and concomitant antidiabetic therapies.

The present findings should also be interpreted within the evolving landscape of lipid-lowering therapy. Although PCSK9 inhibitors such as alirocumab are highly effective in reducing LDL-C levels, their implementation in routine clinical practice may be limited by reimbursement policies and healthcare accessibility. Fogacci et al. demonstrated that many patients eligible for PCSK9 inhibitors according to guideline recommendations remained unable to receive treatment because of reimbursement restrictions (Fogacci et al., 2022). Moreover, the emergence of newer therapies, including inclisiran and bempedoic acid, has expanded the therapeutic options for dyslipidemia management and supports a more individualized approach to lipid lowering. In this context, our findings provide additional real-world evidence supporting the effectiveness of alirocumab in high-risk patients with inadequately controlled LDL-C despite conventional therapy (Saad Alghasab et al., 2024).

The application of artificial intelligence to assess the effectiveness of Alirocumab treatment based on patient characteristics, comorbidities, and medication history represents one of the key contributions of this study. The obtained results demonstrate that machine learning models can provide useful insights into predicting LDL improvement; however, the moderate predictive performance observed across all models (accuracy ranging from 0.50 to 0.63) suggests that treatment response is influenced by a complex combination of clinical and pharmacological factors rather than a single dominant predictor. This interpretation is supported by the correlation analysis, which showed mostly weak to moderate relationships between individual variables and LDL improvement, indicating that the response to Alirocumab is likely driven by multiple interacting factors.

Our findings are consistent with previous studies that have highlighted the potential role of artificial intelligence in personalized lipid management. Yepez et al. demonstrated that machine learning-based models, particularly the Random Forest Classifier, may serve as valuable clinical decision-support tools for optimizing LDL-C management in patients with cardiovascular risk. By integrating patient-specific risk factors, these models can support individualized therapy selection, improve LDL-C target attainment, and potentially reduce therapeutic inertia in real-world clinical practice (Yepez et al., 2025). Nevertheless, further validation in larger and more diverse populations is required before such models can be routinely implemented in clinical care.

In the current study, among the evaluated algorithms, the Support Vector Machine (SVM) achieved the highest predictive performance, with the best balance between accuracy, precision, recall, and F1-score. This finding suggests that nonlinear relationships exist between patient characteristics and treatment response and that kernel-based learning methods may be more effective than traditional linear approaches in capturing these patterns. The comparable performance of ensemble methods such as Random Forest and XGBoost further supports the notion that LDL improvement depends on complex interactions among demographic characteristics, lifestyle factors, comorbidities, and concurrent medications.

Class-wise analysis revealed that most models were more effective in identifying patients who achieved LDL improvement than those who did not. For example, the SVM model achieved a recall of 0.74 for responders compared with 0.47 for non-responders. Similarly, Logistic Regression and Gaussian Naïve Bayes demonstrated high sensitivity toward LDL improvement but showed substantial difficulty in identifying non-responders, resulting in a high number of false-positive predictions. This tendency was evident across the confusion matrices, where most models exhibited a bias toward predicting the “Improve” class. From a clinical perspective, such behavior may be beneficial when the goal is to identify patients who are likely to benefit from Alirocumab therapy. However, the relatively low specificity indicates that some patients may be incorrectly classified as responders, highlighting the need for larger and more balanced datasets to improve discrimination between responders and non-responders.

Overall, these findings show that AI can be a useful decision-support tool for predicting LDL response to Alirocumab, but not a replacement for clinical judgment. The web-based LDL improvement predictor is a practical example of how such models could support clinicians in making more personalized treatment decisions.

However, better performance will likely require larger and more balanced datasets, along with additional clinical variables, biomarkers, and possibly genetic information. Also, the overall moderate accuracy and class imbalance observed suggest that data quantity and feature richness remain limiting factors. The current dataset (n = 206) provides limited variability, which likely constrained model generalization. The skewed performance toward predicting LDL improvement also reflects potential bias arising from unequal class representation or overlapping feature distributions.

Furthermore, model performance was evaluated using a single 80/20 train-test split on a relatively small dataset comprising 206 patients. While this approach provides an initial estimate of predictive capability, it does not offer the robustness required for clinical model development. More rigorous validation approaches, such as k-fold cross-validation or bootstrapping, were not performed and should be considered in future work.

To conclude, this study validates the use of Alirocumab in real-life settings, for improving lipid levels in patients who have high LDL-C levels resistant to conventional treatment. AI analytics add innovation to Alirocumab’s clinical decision pathway strengthening the case for integrating predictive analytics into models of personalized healthcare. While our findings align with controlled trial evidence, the retrospective single-center design is a limitation. The comparative analysis highlights the potential of AI-driven models to support personalized treatment evaluation for lipid-lowering therapies such as Alirocumab. The observed trends emphasize that model performance is closely tied to data size, feature representation, and class balance. Future research should aim to expand the dataset, incorporate biochemical and genetic biomarkers, and apply ensemble optimization or hybrid deep learning frameworks to improve predictive accuracy and interpretability.

A limitation of the present study is that the retrospective nature of the study limited the availability of detailed information regarding recurrent vascular events while taking maximally tolerated statin-based therapy; therefore, patients could not be further classified according to the extreme cardiovascular risk category proposed in the updated ESC/EAS guidelines for the management of dyslipidaemias (Mach et al., 2025). In addition, the logistic regression analyses were exploratory and unadjusted for potential confounders such as statin intensity, BMI and medication adherence. Therefore, the observed associations between concomitant medications and lipid target achievement should not be interpreted as causal and may be influenced by residual confounding. Further prospective studies with larger sample sizes and multivariable-adjusted analyses are required incorporating AI tools to build these findings, ensuring more precise and routine lipid management in everyday clinical practice.

## 5 Conclusion

The current retrospective study confirms clinical efficacy of Alirocumab in the substantial improvement of lipid parameters, especially LDL-C, in very high cardiovascular risk patients not responding to standard lipid-lowering treatment. The findings are consistent with the large-scale randomized trials, which support the role of PCSK9 inhibitors in clinical practice. Patient-characteristics and treatment co-administration effect on the lipid target achievement were identified providing important information for personalized treatment strategies. Furthermore, the AI-based predictive models demonstrated moderate to good accuracy in forecasting Alirocumab treatment response, highlighting the potential applicability of machine learning techniques in supporting clinical decision-making and personalized therapy.

## Data Availability

The raw data supporting the conclusions of this article will be made available by the authors, without undue reservation.
